# Praxis-BGM: clustering of omics data using semi-supervised transfer learning for Gaussian mixture models via natural-gradient variational inference

**DOI:** 10.1093/bioinformatics/btag395

**Published:** 2026-06-17

**Authors:** Qiran Jia, Jesse A Goodrich, David V Conti

**Affiliations:** Division of Biostatistics and Health Data Science, Department of Population and Public Health Sciences, Keck School of Medicine, University of Southern California, Los Angeles, CA 90033, United States; Division of Environmental Health, Department of Population and Public Health Sciences, Keck School of Medicine, University of Southern California, Los Angeles, CA 90033, United States; Division of Biostatistics and Health Data Science, Department of Population and Public Health Sciences, Keck School of Medicine, University of Southern California, Los Angeles, CA 90033, United States; Department of Biostatistics and Informatics, Colorado School of Public Health, Aurora, CO 80045, United States

## Abstract

**Motivation:**

High-dimensional omics data are typically measured on limited sample sizes, which challenges model-based clustering methods such as Gaussian mixture models (GMMs), often leading to instability and poor generalization under complex mixture structures. To address these limitations, we developed Praxis-BGM, a natural-gradient variational inference framework for GMMs. Praxis-BGM enables semi-supervised transfer learning by incorporating an informative prior GMM estimated from large-scale reference data with robust cluster structures. The prior model can encode cluster-specific means, covariance structures, and structural connectivity patterns, and is updated using the target data with variational inference to improve clustering in small-sample settings.

**Results:**

Using the Variational Online Newton (VON) algorithm, we derived natural-gradient updates for the standard parameters of GMMs. Implemented in the Python library JAX for accelerator-oriented computation, Praxis-BGM is computationally efficient and scalable. Across extensive simulations and two real-world applications—breast cancer bulk transcriptomics for subtype recovery and single-cell transcriptomics for cross-platform cell-type label transfer—Praxis-BGM improves posterior clustering performance, stability, and biological interpretability, even when priors are partially mismatched.

**Availability and implementation:**

Praxis-BGM is freely available at https://github.com/ContiLab-usc/Praxis-BGM, and an archival version is available on Zenodo at https://doi.org/10.5281/zenodo.19657680.

## 1 Introduction

Omics data, such as transcriptomics, metabolomics, and proteomics, offer potential insight into human health and disease. Since omics data capture a vast array of molecular parameters, the complex patterns within these measurements can reveal distinct disease mechanisms, variations in disease risk, or heterogeneous survival probabilities (Mo *et al.* 2013, Rappoport and Shamir 2018). Unsupervised clustering methods, such as Gaussian Mixture Models (GMMs), infer underlying patterns and assign data points to latent clusters, playing a crucial role in omics analyses for data exploration, disease risk stratification, cell-type identification, and biomarker discovery (Fraley and Raftery 2002, Mo *et al.* 2013). However, complex correlations, high dimensionality, and limited sample sizes (HDLSS) in many cohort studies pose substantial challenges for statistical analysis (Mo *et al.* 2013, Rappoport and Shamir 2018, Wade 2023, Zhao *et al.* 2025).

As a burgeoning concept, statistical transfer learning offers an effective strategy for addressing the HDLSS challenges by leveraging a related source domain to improve modeling and inference in a target domain, especially in generalized linear model (GLM)-based settings (Pan and Yang 2010, Li *et al.* 2022, Suder *et al.* 2025). Existing transfer-learning extensions of GMMs have mainly focused on unsupervised settings, in which all available datasets are jointly clustered. One example is a multi-task GMM framework that jointly learns multiple related GMMs by leveraging shared parameters across them to improve robustness to outlier datasets (Tian *et al.* 2026). Distributed transfer GMMs, in contrast, learn node-specific clustering models while exchanging model information across decentralized sites to improve clustering without sharing raw data (Wang *et al.* 2023). While useful, these approaches do not directly address the setting in which the source domain is well characterized by robust clusters, often with interpretable annotations, and a reference GMM can therefore be reliably fitted or constructed, such as when a large biobank or consortium dataset with cluster annotations is used to guide clustering in a smaller, more specialized clinical study. Bayesian inference provides a natural mechanism for transfer learning in this setting by encoding source-domain knowledge into informative priors (Suder *et al.* 2025, Wycoff *et al.* 2025). Accordingly, we propose Praxis-BGM, a semi-supervised Bayesian transfer-learning method for GMMs, in which the source-derived GMM is used as an informative prior for inference in the target domain. Building on recent advances in natural gradient variational inference (NGVI) for mixtures of minimal exponential-family distributions (Khan and Nielsen 2018, Lin *et al.* 2019, Mahdisoltani 2021), Praxis-BGM effectively allows the source-derived prior model to regularize the posterior while still adapting to the observed target domain.

**Figure 1:**
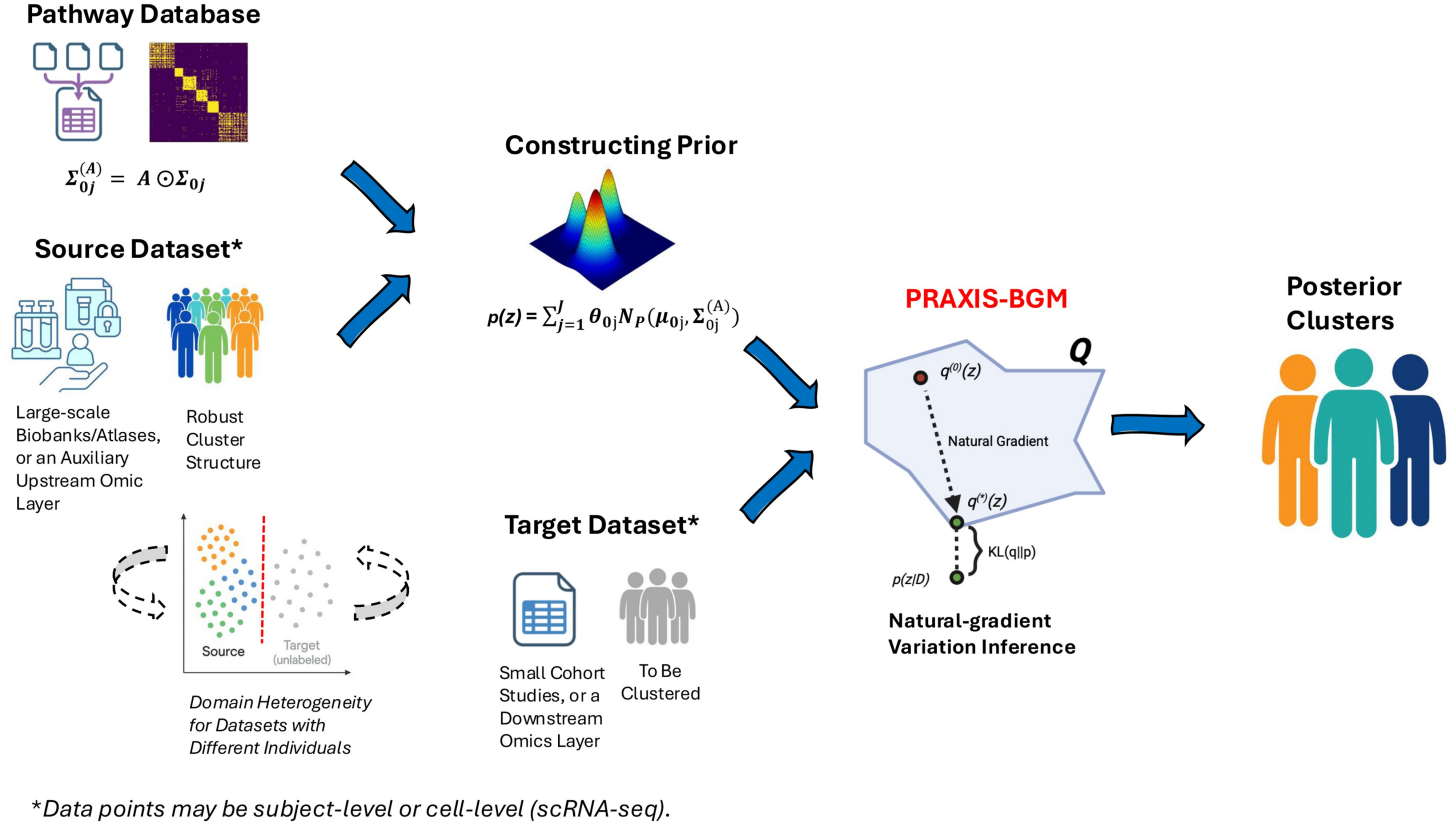
Overview of the Praxis-BGM framework. Informative prior construction is performed using a source dataset with a robust cluster structure, followed by Bayesian transfer learning for Gaussian Mixture Models via natural-gradient variational inference in the target dataset. The source dataset for informative prior construction can originate either from a separate dataset measured on different samples (e.g. large-scale biobanks or atlases) for cross-dataset transfer learning, or from an informative upstream omic layer measured on the same samples (e.g. using RNA-seq to inform miRNA) for multi-omics in-serial integration.

Figure 1 provides an overview of the Praxis-BGM workflow, illustrating prior construction from a source domain and NGVI-based update in the target domain. In this setting, the source domain is assumed to contain robust cluster structures or known curated cluster labels, and ideally to be annotated with meaningful biological interpretations (e.g. cancer subtypes). The prior GMM model constructed from the source domain can encode cluster-specific means, covariance structures, and structural connectivity patterns informed by pathway databases. To the best of our knowledge, no existing Bayesian framework has been proposed for semi-supervised transfer learning in GMMs under labeled reference datasets. This provides a principled transfer mechanism that complements existing GLM-based transfer learning strategies (Li *et al.* 2022). We also showed that priors can be constructed not only from a source dataset in the same feature space with different samples, but also from an auxiliary omic layer measured on the same samples, thereby enabling multi-omics in-serial integration, as explored in previous studies but within different modeling frameworks (Goodrich *et al.* 2024, Zhao *et al.* 2025).

We applied Praxis-BGM to breast cancer subtyping across transcriptomic datasets to transfer subtype structure from a source cohort to a target cohort. We also applied the proposed method to the task of label transfer across single-cell RNA-seq technologies (i.e. sequencing platforms), such as inDrop (droplet-based) and CEL-seq, using pancreatic single-cell RNA-seq data, where transfer and inference were performed in the latent embedding space. In both applications, ground-truth labels were either fully or partially available for the target data, allowing for a direct validation of Praxis-BGM’s superior performance in transferring prior knowledge and guiding clustering.

The rest of the paper is organized as follows: Section 2 details the proposed method under the NGVI framework. Section 3 introduces the real-world data for applications and outlines the experimental settings. Section 4 reports the results, demonstrating the strong and consistent advantages of Praxis-BGM. We also present extensive simulation results evaluating the effectiveness of Praxis-BGM in transferring cluster structure via individual components of informative priors, as well as jointly, across diverse signal-to-noise regimes and source–target discrepancies in Data 1 and 2, available as supplementary data at *Bioinformatics* online. Overall, these simulation results demonstrate that Praxis-BGM achieves efficient, robust, and consistently superior performance relative to competing methods, even under challenging and partially misspecified conditions. Finally, Section 5 concludes the paper by summarizing the findings and limitations, as well as outlining future research directions.

## 2 Materials and methods

Let $z\in R^{P}$ be a random variable following a GMM (i.e. a mixture of multivariate Gaussian distributions), with *J* components. Its marginal density is


(1) $$p(z)=\sum_{j=1}^{J}\theta_{j}N_{P}\left(z|\mu_{j},\Sigma_{j}\right),\;\text{such}\;\text{that}\sum_{j=1}^{J}\theta_{j}=1,$$

where $\theta_{j}$ is the mixing weight for component *j*, $\mu_{j}\in R^{P}$ is the corresponding mean vector, and $\Sigma_{j}\in S_{++}^{P}$ is the corresponding symmetric positive definite (SPD) covariance matrix.

### 2.1 Priors from the source data in the same feature space as the target data

Consider a source dataset $D_{0}$, measured in the same *P*-dimensional feature space as the target dataset D, that is available from a related population. We utilize $D_{0}$ to construct informative priors for *z*. This yields a Bayesian transfer-learning formulation in which the source data inform the prior distribution $p(z|D_{0})$, and the target data D updates this prior through Bayes’ rule (Suder *et al.* 2025). Specifically,


(2) $$p(z|D,D_{0})\propto p(D|z)p(z|D_{0}).$$

The normalizing constant $p(D|D_{0})=\int p(D|z)p(z|D_{0})dz$ is generally intractable. Therefore, in a two-stage procedure, we first estimate the source-informed prior distribution $p(z|D_{0})$ and then use variational inference to approximate the posterior $p(z|D,D_{0})$ by maximizing a tractable lower bound on the log marginal likelihood, namely the evidence lower bound (ELBO) (Blei *et al.* 2017).

Specifically, we parameterize the prior $p(z|D_{0})$ as a GMM with parameters $\omega ={\left\{\theta_{0j},\mu_{0j},\Sigma_{0j}\right\}}_{j=1}^{J}$, representing the distribution learned from $D_{0}$. When $D_{0}$ includes cluster labels $X_{0}$, we can directly use cluster-specific summary statistics or discriminant analysis to derive ω (Fraley and Raftery 2002). In the absence of labels, a standard GMM can be used to estimate the prior mixture distribution, with the optimal number of clusters selected using standard criteria such as the Bayesian Information Criterion (BIC) (Fraley and Raftery 2002, Wade 2023). It is often useful to inflate the variance of the source-informed prior, as the distribution of *z* may differ substantially across domains, to improve robustness (Suder *et al.* 2025). Specifically, for each component *j*, we replace $\Sigma_{0j}$ by $c\Sigma_{0j}$ with c>1. The prior cluster-specific covariance matrices $\Sigma_{0j}$ can optionally be adjusted by additional prior knowledge to encourage sparsity as


(3) $$\Sigma_{0j}^{\left(A\right)}=A_{j}⊙\Sigma_{0j},$$

where the sparsity–imposing matrix $A_{j}\in {\left\{0,1\right\}}^{P\times P}$ is symmetric with unit diagonal $\left(A_{j,pp}=1,\;\forall p\right)$, and is derived from grouping information, such as that available from existing pathway databases to encode element-wise structural modulation (Thomas *et al.* 2009). The matrix $A_{j}$ shapes the prior covariance matrix via a Hadamard mask as shown in Equation (3).

### 2.2 Priors from an auxiliary omic layer measured on the same samples as the target data

We also allow for constructing informative priors from an auxiliary omic layer collected on the same samples as D. Let $D^{\left(O\right)}$ denote an alternative omic layer (e.g. DNA methylation) measured on the same subjects who provide the target data D (e.g. transcriptomics). When cluster or class labels $X^{\left(O\right)}$ are available or can be reliably inferred from $D^{\left(O\right)}$, we construct a prior distribution $p(z|D^{\left(O\right)})$ parameterized by $\omega ={\left\{\theta_{0j},\mu_{0j},\Sigma_{0j}\right\}}_{j=1}^{J}$, where the parameters are obtained from discriminant analysis or empirical class-conditional summary statistics in the target feature space conditioned on $X^{\left(O\right)}$ (Fraley and Raftery 2002). This enables using a biologically related but distinct omic layer, measured on the same subjects, to derive the prior distribution for the latent variable *z* representing upstream cluster-structure signals, in line with in-serial integration strategies for multi-omics data (Goodrich *et al.* 2024, Zhao *et al.* 2025).

### 2.3 Variational inference with informative priors

Given informative priors derived from either the source data $D_{0}$ or the auxiliary omic layer $D^{\left(O\right)}$, denoted collectively by $p_{\omega }(z)$ for simplicity, we approximate the posterior with a surrogate finite mixture of multivariate Gaussian distributions $q_{\phi }(z|D)$ with $\phi ={\left\{\theta_{j},\mu_{j},\Sigma_{j}\right\}}_{j=1}^{J}$ using a NGVI framework (Khan and Nielsen 2018, Lin *et al.* 2019). Given priors $p_{\omega }(z)$ and target data D, we seek a variational distribution that maximizes the ELBO,


(4) $$L=E_{q_{\phi }(z|D)}[\text{log}\;p(D|z)]-D_{\text{KL}}[q_{\phi }(z|D)\|p_{\omega }(z)].$$

Maximizing L encourages $q_{\phi }(z|D)$ to explain the target mixture data D well, while remaining regularized toward the prior $p_{\omega }(z)$. In general, NGVI is a first-order optimization method for maximizing L of minimal exponential-family (EF) distributions that replaces the standard Euclidean gradient with the natural gradient, thereby accounting for the information geometry of the natural parameter space (Khan and Nielsen 2018). Natural gradient yields parameterization invariance and often significantly improves convergence efficiency and stability (Amari 1998, Khan and Nielsen 2018). Specifically, NGVI updates the natural parameter $\lambda_{z}$ by scaling the standard Euclidean gradient with the inverse Fisher information matrix (FIM): $\lambda_{z}^{\left(t+1\right)}=\lambda_{z}^{\left(t\right)}+\beta F^{-1}\left(\lambda_{z}^{\left(t\right)}\right)\nabla_{\lambda_{z}}L\left(\lambda_{z}^{\left(t\right)}\right),$ where β is the step size. Under high-dimensional settings, direct inversion of the FIM is computationally burdensome. For minimal EF variational distributions, one can avoid this explicit evaluation by working in the expectation-parameter space $m_{z}$, using the duality between natural and expectation parameters (Khan and Nielsen 2018). Under this duality, the natural gradient for $\lambda_{z}$ can thus be expressed as the ordinary gradient with respect to $m_{z}$: $F^{-1}\left(\lambda_{z}^{\left(t\right)}\right)\nabla_{\lambda_{z}}L\left(\lambda_{z}^{\left(t\right)}\right)=\nabla_{m_{z}}L_{*}\left(m_{z}^{\left(t\right)}\right),$ where $L_{*}$ denotes the ELBO expressed in the expectation-parameter space. $\nabla_{m_{z}}L_{*}\left(m_{z}^{\left(t\right)}\right)$ can be obtained in terms of gradients with respect to the standard parameters (e.g. μ and Σ for Gaussian) by using the chain rule, which in turn yields the corresponding natural-gradient update for standard parameters via the Variational Online Newton (VON) algorithm (Lin *et al.* 2019). NGVI has been extended beyond minimal EF to mixtures of minimal EF distributions, including finite mixtures of multivariate Gaussians (i.e. GMMs), under a minimal conditional EF representation (Lin *et al.* 2019, Mahdisoltani 2021).

We apply NGVI for GMMs with informative priors obtained from the source data, $p_{\omega }(z)$, to facilitate Bayesian transfer learning. We define $L(\lambda_{z})=E_{q_{\phi }(z|D)}[-h(z)],$ and $h(z)=-\sum_{n=1}^{N}\;\text{log}\;p(D_{n}|z)+\text{log}\;\frac{q_{\phi }(z|D)}{p_{\omega }(z)}.$ The VON algorithm is used to efficiently evaluate h(z) by drawing a stochastic sample under $q_{\phi }(z|D)$ and then use it to compute natural gradients for standard parameters ϕ of GMMs (Khan and Nielsen 2018, Lin *et al.* 2019). Specifically, at iteration *t*, we first draw a component *v* and then draw a sample conditioned on the component,


(5) $$v\;∼\;\text{Cat}(\theta^{\left(t\right)}),z_{0}\;∼\;q_{\phi }^{\left(t\right)}(z|j=v),$$

which serves as the anchor point for natural-gradient updates. We then define


(6) $$\begin{array}{c} h(z_{0})=\text{log}\;\left(\sum_{j=1}^{J}\theta_{j}N(z_{0}|\mu_{j},\Sigma_{j})\right)\\ -\text{log}\;\left(\sum_{j=1}^{J}\theta_{0j}N(z_{0}|\mu_{0j},\Sigma_{0j})\right)\\ -\sum_{n=1}^{N}\;\text{log}\;p(D_{n}|z_{0}). \end{array}$$

The stochastic gradient and Hessian of $h(z_{0})$ with respect to $z_{0}$, denoted by $\nabla_{z}h(z_{0})$ and $\nabla_{z}^{2}h(z_{0})$, respectively, are key quantities in the derived NGVI updates for standard parameters ϕ, obtained using Bonnet’s and Price’s theorems as shown in Lin *et al.* (2019):


(7) $$\Sigma_{j}^{-1\;(t+1)}=\Sigma_{j}^{-1\;(t)}+\beta \delta_{j}(z_{0})\nabla_{z}^{2}h(z_{0}),$$

(8) $$\mu_{j}^{\left(t+1\right)}=\mu_{j}^{\left(t\right)}-\beta \delta_{j}(z_{0})\Sigma_{j}^{\left(t+1\right)}\nabla_{z}h(z_{0}),$$

(9) $$\begin{array}{c} \;\text{log}\;\frac{\theta_{j}^{\left(t+1\right)}}{\theta_{J}^{\left(t+1\right)}}=\text{log}\;\frac{\theta_{j}^{\left(t\right)}}{\theta_{J}^{\left(t\right)}}-\beta (\delta_{j}(z_{0})-\delta_{J}(z_{0}))h(z_{0}),\\ \;j=1,\ldots ,J-1. \end{array}$$

where


(10) $$\delta_{j}(z_{0})=\frac{N(z_{0}|\mu_{j}^{\left(t\right)},\Sigma_{j}^{\left(t\right)})}{\sum_{\ell =1}^{J}\theta_{\ell }^{\left(t\right)}N(z_{0}|\mu_{\ell }^{\left(t\right)},\Sigma_{\ell }^{\left(t\right)})}.$$

We summarize Praxis-BGM (prior-augmented NGVI for GMMs) in Algorithm 1. We initialize the variational parameters ϕ using the prior parameters ω to facilitate alignment between the target-domain cluster labels and the prior structure, thereby enhancing interpretability. Praxis-BGM is implemented in Python using JAX, enabling accelerator-friendly computation on GPUs and TPUs, as well as just-in-time compilation for scalable and numerically stable NGVI (Bradbury *et al.* 2018).

Algorithm 1 Praxis-BGM: Prior-augmented Natural Gradient Variational Inference for Gaussian Mixture Models

**Require:** Target data $D={\left\{D_{n}\right\}}_{n=1}^{N}$; source data $D_{0}$ or $D^{\left(O\right)}$ with labels $X_{0}$ or $X^{\left(O\right)}$defining prior parameters $\omega =(\theta_{0},{\left\{\mu_{0j},\Sigma_{0j}\right\}}_{j=1}^{J})$, where $\left\{\Sigma_{0j}\right\}$ and $\theta_{0}$ may be specified only if $\left\{\mu_{0j}\right\}$ is provided; structural sparsity masks $\left\{A_{j}\right\}$.

**Require:** Stepsize β, number of epochs *T*, covariance-scaling constant *c*, jitter $\tau =10^{-9}$.

1: **Construct working prior parameters**  $\omega^{⋆}$**:** 

2: **for**  j=1,…,J  **do** 

3:  $\mu_{0j}^{⋆}\leftarrow \{\begin{array}{cc} \mu_{0j}, & \text{if}\;\text{provided}\\ 0_{P}, & \text{otherwise} \end{array}$

4:  $\Sigma_{0j}^{⋆}\leftarrow \{\begin{array}{cc} (A_{j}⊙\Sigma_{0j})c+\tau I_{P}, & \text{if}\;\text{provided}\\ cI_{P}, & \text{otherwise} \end{array}$

5: **end for** 

6: $\theta_{0}^{⋆}\leftarrow \{\begin{array}{cc} \theta_{0}, & \text{if}\;\text{provided}\\ J^{-1}1_{J}, & \text{otherwise} \end{array}$

7: **Initialize variational parameters**  ϕ**:** 

8: **if** prior means $\left\{\mu_{0j}\right\}$ are provided **then** 

9:  $\mu_{j}\leftarrow \mu_{0j}^{⋆}$, $\Sigma_{j}\leftarrow \Sigma_{0j}^{⋆}$, $\theta \leftarrow \theta_{0}^{⋆}$

10: $\rho_{j}\leftarrow \;\text{log}(\theta_{j}/\theta_{J})$

11: **else** 

12:  Fit an optimal *BayesianGaussianMixture* Model (Pedregosa *et al.* 2011) on D to obtain initial $\mu_{j}$, $\Sigma_{j}$

13:  $\theta \leftarrow J^{-1}1_{J}$ and initialize $\rho_{j}$ accordingly

14: **end if** 

15: **for**  t=0,…,T−1  **do** 

16:  Sample anchor $z_{0}$ from $q_{\phi }$ using Eq. (5)

17:  Compute $\delta_{j}(z_{0})$ from Eq. (10) and the global term $h(z_{0})$ from Eq. (6)

18:  **for**  j=1,…,J  **do** 

19:   Update $\Sigma_{j}^{-1}$, $\mu_{j}$, and $\rho_{j}$ following the VON update defined in Eqs (7)–(9), respectively.

20:  **end for** 

21:  $\theta \leftarrow \text{softmax}([\rho_{1},\ldots ,\rho_{J-1},0])$

22: **end for** 

23: **Output:** Posterior parameters ϕ, and cluster responsibilities $\gamma_{nj}$.

## 3 Evaluation with real-world data

We demonstrated the effectiveness of Praxis-BGM in transferring prior cluster structural information to improve clustering performance and enhance clinical relevance compared with standard clustering and classification methods across two real-world data applications.

### 3.1 Breast cancer bulk transcriptomics

The Cancer Genome Atlas Breast Invasive Carcinoma (TCGA-BRCA) and the Molecular Taxonomy of Breast Cancer International Consortium (METABRIC) are two breast cancer genomic cohorts (Curtis *et al.* 2012, Network 2012), each profiling the transcriptomic expression of ∼20 000 genes (bulk RNA-seq data) and accompanied by clinical and survival information. TCGA-BRCA, downloaded from the R package curatedTCGAData (Ramos *et al.* 2020), includes PAM50 subtype labels for 500 subjects. On the other hand, METABRIC, accessed through cBioPortal (Cerami *et al.* 2012, Gao *et al.* 2013, de Bruijn *et al.* 2023), provides hormone-receptor status, which is a well-established surrogate for intrinsic subtypes (Perou *et al.* 2000, Parker *et al.* 2009). For METABRIC, we constructed surrogate subtype labels by mapping clinical receptor status to their closest intrinsic subtype counterparts. Specifically, ER+/HER2—cases were categorized as Luminal A-like, ER+/HER2+ as Luminal B-like, ER–/PR–/HER2+ as HER2-enriched-like, and ER–/PR–/HER2– as Triple-negative, used here as a surrogate for Basal-like subtype given their strong overlap (Perou *et al.* 2000). These labels are interpreted as approximate clinical proxies for breast cancer subtypes, and we used them to derive related but not fully accurate informative priors for transfer-learning-based clustering. For TCGA-BRCA, we utilized the RNA-based PAM50 labels available as ground-truth, noting that 560 subjects (52.83%) in the expanded cohort lack these formal molecular annotations.

The RNA-seq data of METABRIC were used as the labeled source dataset to derive priors for Praxis-BGM, with the RNA-seq data of TCGA-BRCA serving as the unlabeled target dataset. We hypothesized that Praxis-BGM with informative priors estimated from METABRIC would yield clusters in TCGA-BRCA that better align with PAM50 subtypes and exhibit stronger prognostic relevance than unsupervised clustering or supervised prediction across datasets alone. After filtering incomplete records, 1980 METABRIC and 1060 TCGA-BRCA samples remained. We restricted the analysis to genes shared between METABRIC and TCGA-BRCA and used log_2_-transformed expression values. The top 1000 most variable genes were selected from METABRIC based on an elbow criterion applied to the ranked gene-variance plot, thereby excluding low-information genes. METABRIC and TCGA-BRCA were then subset to the selected gene set and standardized before downstream analysis. For each METABRIC subtype, we computed empirical means and covariance matrices to form informative priors.

For performance evaluation, Praxis-BGM clusters were compared with the unsupervised Bayesian Gaussian Mixture Models (BGM), a VI-based GMM method with uninformative priors with number of clusters *J *= 4 (Pedregosa *et al.* 2011), and supervised classifiers trained on the source data and applied to the target data, which include multinomial logistic regression, Linear Discriminant Analysis (LDA) (Fraley and Raftery 2002), and eXtreme Gradient Boosting (XGBoost) (Chen and Guestrin 2016) tuned via cross-validation. Estimated cluster labels in TCGA-BRCA were evaluated by Adjusted Rand Index (ARI) and accuracy with PAM50 labels, and were further evaluated as predictors in separate age-adjusted Cox proportional hazards models for five-year survival. Bayes factors (BFs) for feature importance in driving breast cancer subtype differentiation (method detailed in Data 3.1, available as supplementary data at *Bioinformatics* online) were computed to identify subtype-driven genes, followed by functional enrichment analysis using Gene Ontology (GO) Biological Process (Ashburner *et al.* 2000, Consortium 2026) and Human Molecular Signatures Database (MSigDB) Hallmark gene sets (Liberzon *et al.* 2015) via GSEApy (Fang *et al.* 2023).

We next propagated the estimated RNA-seq Praxis-BGM clusters as priors for clustering of the microRNA (miRNA) layer in the same TCGA-BRCA cohort, enabling a second Bayesian update of the GMM while achieving in-serial multi-omics integration (Goodrich *et al.* 2024, Zhao *et al.* 2025). Biologically, miRNA captures regulatory signals not fully reflected by RNA-seq expression alone and may provide complementary information for breast cancer subtyping (Bertoli *et al.* 2015). This in-serial analysis follows Section 2.2 and the analysis method is detailed in Data 3.4, available as supplementary data at *Bioinformatics* online.

### 3.2 Cross-platform label transfer in pancreatic scRNA-seq

When analyzing single-cell RNA sequencing (scRNA-seq) data, a key initial task is to annotate cell types for clusters of cells. The increasing availability of annotated reference datasets has led to numerous label-transfer methods (Pasquini *et al.* 2021). Among them, scANVI (Xu *et al.* 2021), a semi-supervised extension of scVI (Lopez *et al.* 2018), is an effective approach that jointly models labeled reference and unlabeled query data. The Scanpy framework (Hao *et al.* 2021) also provides K-Nearest Neighbors (KNN)-based label-transfer functionality by integrating principal component (PC) embeddings across datasets, called ingest, and heterogeneity-correction methods, such as ComBat-seq (Zhang *et al.* 2020) and Scanorama (Hie *et al.* 2024), can be paired with KNN classifiers for label transfer.

Label transfer naturally aligns with the goal of Praxis-BGM, which performs clustering guided by cell-type–specific priors. We used the human pancreas scRNA-seq atlas assembled in Luecken *et al.* (2022), a benchmark dataset of 16 382 cells annotated into 14 cell types across four platforms (inDrop, CEL-Seq, Smart-seq, Fluidigm C1), chosen for its heterogeneous batch structure. After quality control, cells with zero total counts were removed. Expression values were then log_2_-transformed, and the top 2000 highly variable genes (HVGs) were selected using Scanpy based on standardized gene-level variance. Principal component analysis (PCA) was performed on the selected HVGs, and the first 100 PCs were retained for downstream analysis. Cells from inDrop (52.3%) served as the reference dataset, while cells from the remaining platforms (47.7%) formed the query dataset. Ground-truth annotations were available for all cells and were used only for evaluation on the query subset.

For Praxis-BGM, we computed cell-type–specific mean priors in the PC space corrected by ComBat-seq from the reference data and applied them to guide clustering on the query data. We benchmarked Praxis-BGM against scANVI, Scanpy’s ingest function, and integration-based methods (ComBat-seq and Scanorama) followed by KNN classification. Notably, Praxis-BGM and scANVI use the reference information differently: Praxis-BGM incorporates priors directly into cluster inference on the query set, whereas scANVI learns a shared latent embedding for both datasets before label prediction. For computational time comparisons, we measured only the cost of heterogeneity correction and inference, excluding embedding computation.

## 4 Results

### 4.1 Praxis-BGM enhances breast cancer subtype recovery and clinical relevance in TCGA-BRCA

Our results show that, by leveraging informative priors derived from METABRIC, Praxis-BGM improved both subtype concordance and clinical interpretability relative to unsupervised clustering and supervised classification approaches applied to TCGA-BRCA RNA-seq data.

**Figure 2:**
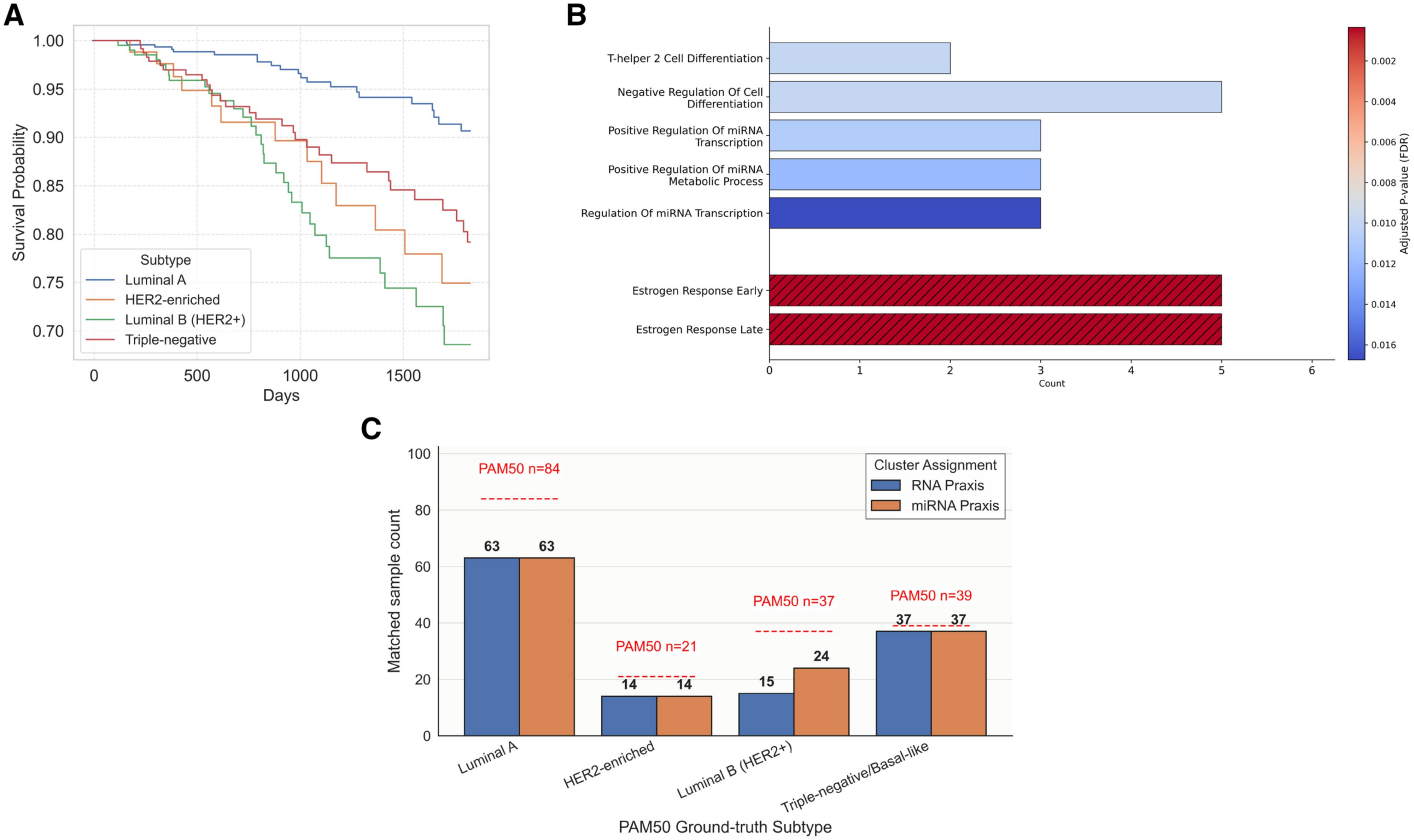
(A) Kaplan–Meier curve comparing survival across Praxis-BGM clusters annotated by subtypes; (B) pathway enrichment analysis of the non-indeterminate genes (BF >  $10^{0.5}$). The solid bars represent pathways from the Gene Ontology (GO) Biological Process database, whereas the hatched bars represent Human Molecular Signatures Database (MSigDB) Hallmark gene sets; (C) subtype-specific concordance of RNA- and miRNA-based Praxis-BGM clusters with PAM50 ground-truth labels.

Table 1 presents the ARI and accuracy results for concordance with ground-truth PAM50 subtype labels for Praxis-BGM and other benchmark methods on TCGA-BRCA RNA-seq data. BGM clusters captured partial concordance with the PAM50 molecular subtypes (ARI = 0.392), indicating that even without subtype-specific priors, the intrinsic transcriptomic structure of TCGA-BRCA samples reflects moderate underlying subtype-related patterns. For classification benchmark methods, multinomial regression and LDA produced similar subtype concordance (ARI = 0.381 and 0.379, accuracy = 0.654 and 0.650, respectively). XGBoost yielded the lowest ARI (0.351) and accuracy (0.638) among the reported supervised classification methods, suggesting potential overfitting despite the use of 10-fold cross-validation for hyperparameter tuning. Among all methods, Praxis-BGM showed the strongest overall agreement with the ground-truth PAM50 subtype labels (ARI = 0.430; accuracy = 0.714). We then visualized the 5-year survival across Praxis-BGM clusters using Kaplan Meier curves in Fig. 2a. The Kaplan–Meier curves show clear survival heterogeneity across the inferred Praxis-BGM clusters. The posterior Praxis-BGM cluster corresponding to Luminal A has the best prognosis, whereas the cluster corresponding to Luminal B (HER2+) has the poorest survival with the steepest decline. Overall, Praxis-BGM clusters yield the strongest survival stratification under the age-adjusted Cox proportional hazards model for 5-year survival (LRT statistic =57.79, $p-\text{value}=8.460\times 10^{-12}$). The Cox model using BGM clusters is likewise significant (LRT statistic =45.121, $p-\text{value}=3.752\times 10^{-9}$) but less pronounced, whereas all three classification methods show weaker significance in their corresponding Cox models. Taken together, these findings show that Praxis-BGM clusters not only recover established subtype structures but also provide superior prognostic stratification beyond benchmark unsupervised and supervised methods through Bayesian transfer learning. As for runtime, although BGM achieved the fastest CPU runtime, Praxis-BGM remained computationally efficient and benefited from TPU acceleration.

**Table 1:** Benchmarking clustering and classification methods on TCGA-BRCA RNA-seq data by assessing the ARI and accuracy with ground-truth PAM50 subtype labels, restricted to the 500 subset of samples with available subtype annotations, and by evaluating prognostic value using the likelihood-ratio test (LRT) statistic and the corresponding *P-*value from age-adjusted Cox proportional hazards models for 5-year survival, and reporting runtime with the corresponding hardware.

Method	ARI	Accuracy	LRT Stat (*P*-value)	Time (s)/hardware
Praxis-BGM	**0.430**	**0.714**	**57.787 (** $8.460\times 10^{-12}$ **)**	6.04/V6e TPU
BGM	0.392	-	45.121 ($3.752\times 10^{-9}$)	**1.77**/CPU
Multinomial Regression	0.381	0.654	31.711 ($2.191\times 10^{-6}$)	2.24/CPU
LDA	0.379	0.650	33.661 ($8.744\times 10^{-7}$)	11.08/CPU
XGBoost	0.351	0.638	30.556 ($3.771\times 10^{-6}$)	11.82/CPU

Bold values indicate the best performance for each metric.

To identify genes driving breast cancer subtype differentiation, we computed BFs for all genes and identified 39 genes showing at least substantial evidence based on Jeffreys’ scale (Jeffreys 1961). To better interpret the identified clustering-driven genes, Fig. 2b shows the results of the pathway over-representation analysis for genes with at least substantial evidence (BF > $10^{0.5}$). After FDR adjustment, seven pathways were significant. Interestingly, the two estrogen response pathways found in MSigDB are well-established signatures in the progression of breast cancer (Yan *et al.* 2024). Among GO Biological Process, the three miRNA-related significant pathways are also biologically relevant to breast cancer, as aberrant miRNA expression has been associated with tumor initiation, progression, and treatment resistance (Bertoli *et al.* 2015). Although these miRNA-related pathways were discovered based on RNA expression data, their enrichment is consistent with underlying miRNA-RNA regulatory mechanisms. These results suggest that integrating informative priors from METABRIC through Praxis-BGM not only yields more clinically relevant clusters but also improves biological interpretability and downstream analysis, further motivating the in-serial integration of the miRNA layer in the target data.

For the in-serial analysis with miRNA data in TCGA-BRCA, leveraging the posterior RNA-seq Praxis-BGM clusters as priors, the posterior miRNA Praxis-BGM clusters were largely consistent with the RNA-based Praxis-BGM clusters while providing refined stratification that improved alignment with PAM50 labels among subjects with available annotations and miRNA data. Specifically, clustering agreement measured by ARI increased from 0.417 to 0.478. To explore this increase, in Fig. 2c we present the subtype-specific concordance of the RNA- and in-serial miRNA-based Praxis-BGM clusters with PAM50 ground-truth labels. Notably, the Praxis-BGM clusters updated with the miRNA layer correctly assigned nine additional subjects to the Luminal B subtype compared with the RNA-based Praxis clusters. These refined posterior miRNA Praxis-BGM clusters incorporate priors from both the external source dataset METABRIC and the RNA-seq layer of TCGA-BRCA, providing an illustrative example of multi-omics integration through sequential Bayesian update.

### 4.2 Praxis-BGM outperforms existing label transfer methods on cross-platform scRNA-seq

A benchmark of cell-type label transfer methods on cross-platform scRNA-seq data revealed that Praxis-BGM provides superior label transfer accuracy and robustness compared with widely used alternatives.

**Figure 3:**
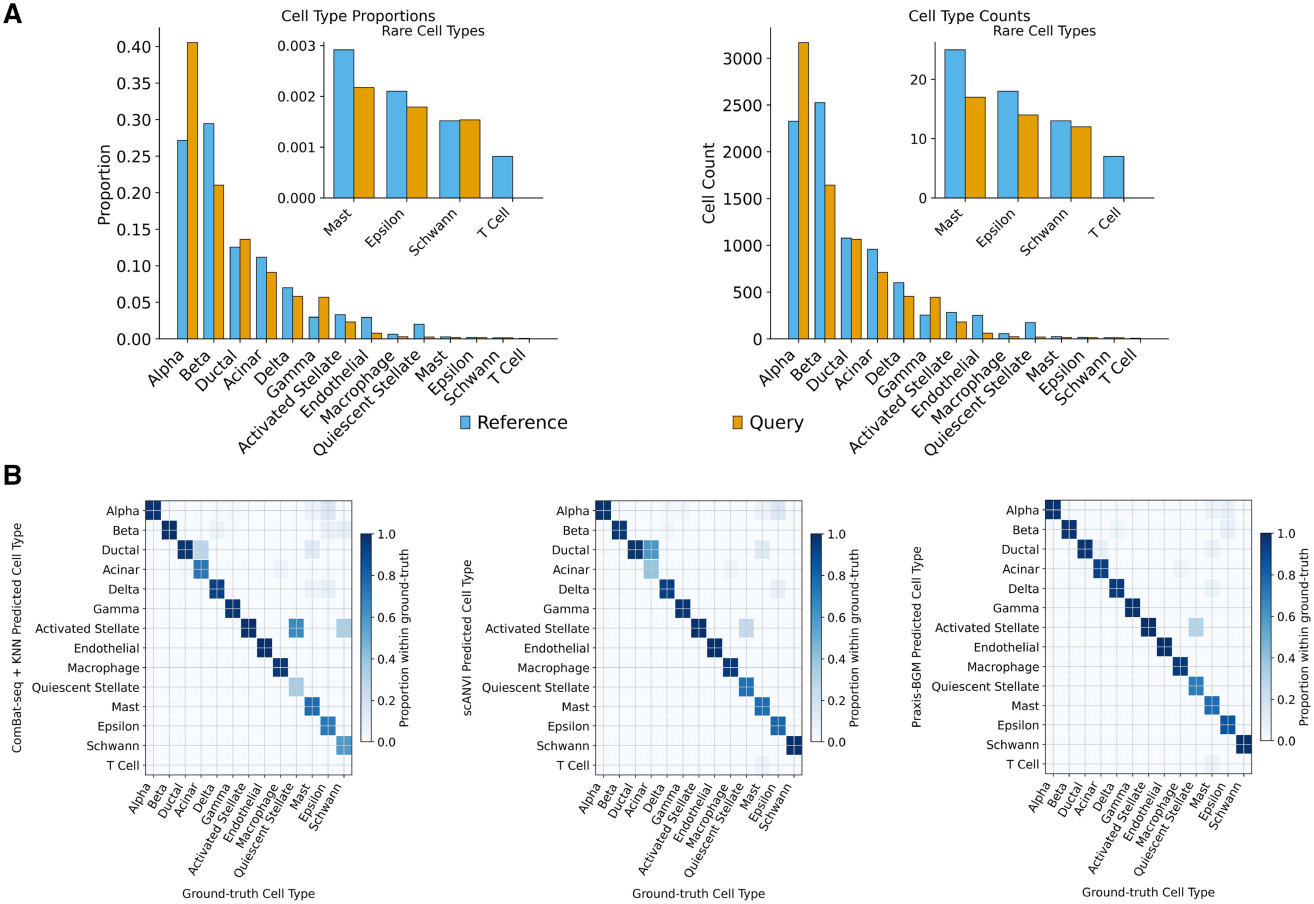
(A) Ground-truth cell type proportions (left) and count (right) distributions for the reference and query datasets. (B) Confusion heatmap comparing predicted cell types to ground-truth annotations in the query data for ComBat-seq + KNN, scANVI, and Praxis-BGM, with color intensity representing the proportion within each ground-truth cell type.

Figure 3a shows the cell-type distributions in the reference and query datasets. The reference dataset contains all cell types present in the query dataset, as well as an additional T-cell population. Table 2 presents the results of benchmark label transfer methods on the query subset. Praxis-BGM and ComBat-seq + KNN substantially outperformed other methods in terms of ARI and accuracy, while also being computationally faster. Notably, Praxis-BGM achieved the highest clustering accuracy and ARI (accuracy = 0.975; ARI = 0.957), better than those of ComBat-seq + KNN and scANVI. Because ComBat-seq + KNN uses the same corrected PC embeddings as Praxis-BGM, the 2.2% improvement in accuracy and 0.024 improvement in ARI further highlight the performance advantage of Praxis-BGM over the deterministic classification method of KNN. Scanpy-ingest and Scanorama + KNN yielded inferior ARI and accuracy, and required longer computation times than Praxis-BGM did. Among all methods, scANVI was the most computationally intensive as it is neural-network-based.

**Table 2:** Benchmarking label transfer methods on pancreas single-cell transcriptomic data by assessing predicted labels using ARI and accuracy against expert annotations, reporting runtime with the corresponding hardware platform, and the dimension-reduced embedding that each method takes.

Method	ARI	Accuracy	Time (s)	Hardware	Embedding
Praxis-BGM	**0.957**	**0.975**	**2.94**	T4 GPU	PCs
ComBat-seq + KNN	0.933	0.953	3.4	CPU	PCs
scANVI	0.912	0.921	33.0	T4 GPU	scVI
Scanorama + KNN	0.913	0.916	15.5	CPU	PCs
Scanpy-ingest	0.896	0.915	10.3	CPU	PCs

Bold values indicate the best performance for each metric.

Praxis-BGM and scANVI relied on different embeddings to perform the label transfer (PCs for Praxis-BGM and scVI representations for scANVI). The improved performance of Praxis-BGM compared with other KNN-based methods that also use PC embeddings suggests a potential advantage of Praxis-BGM in better adapting to settings where the reference and query domains are not fully homogeneous, leveraging variational inference. Figure 3b provides the confusion heatmap comparing predicted cell types to ground-truth annotations of the cell types in the query data. Notably, the annotated reference subset contained 7 T cells while the query subset did not (Fig. 3a). ComBat-seq + KNN and scANVI exhibited pronounced misassignment of Acinar cells to Ductal, while Praxis-BGM did not. All methods showed some degree of confusion between Quiescent and Activated Stellate cells, likely due to their similarity, and all performed somewhat less accurately on the rarer cell types of Mast and Epsilon. Praxis-BGM and scANVI accurately assigned the rare cell type of Schwann, while ComBat-seq + KNN did not. In addition, we conducted an additional sensitivity analysis investigating whether KNN-based benchmark label-transfer methods would benefit from using a smaller and more targeted set of source-derived differentially expressed genes (DEGs) in Data 4.1, available as supplementary data at *Bioinformatics* online, rather than the original workflow based on 2000 HVGs. Overall, more aggressive DEG restriction modestly improved KNN-based benchmark methods, while Praxis-BGM remained the best-performing approach across all methods. We also evaluated a scenario in which the reference dataset contained fewer rare cell types than the query dataset (Data 4.2, available as supplementary data at *Bioinformatics* online). Under this mismatch, Praxis-BGM still outperformed the benchmark methods.

This application demonstrates that Praxis-BGM can leverage cell-type—informative priors to achieve accurate and computationally efficient label transfer between heterogeneous single-cell datasets, making it a practical and scalable alternative to existing methods. Looking ahead, as emerging technologies (e.g. *in situ* spatial transcriptomics) enable more comprehensive characterization of rare cell types, increasingly comprehensive reference datasets can be used for Praxis-BGM. Capable of addressing platform heterogeneity, Praxis-BGM is also especially useful for transferring well-annotated scRNA-seq labels to spatial RNA-seq data, where direct cell-type identification remains challenging.

## 5 Discussion

In this paper, we introduced Praxis-BGM, a semi-supervised GMM clustering method based on NGVI that facilitates transfer learning. The source data are assumed to have curated cluster labels, which, if not already available, can be obtained by fitting an unsupervised GMM with an appropriate model selection procedure and subsequently annotating the labels through biological interpretation. Relying on the source data, Praxis-BGM provides a highly flexible framework for Bayesian transfer learning, encoding source knowledge in a prior GMM and adapting it to the target data through variational inference. One or any combination of cluster-specific parameters in the prior GMM can be specified, including the mean vector (prior belief in differences in expression levels across clusters), covariance matrix (prior belief in differences in variability and correlation patterns across clusters), and weights (prior belief in differences in cluster prevalence). In addition, overall or cluster-specific sparsity-inducing matrices informed by pathway and ontology databases can be incorporated to encode feature connectivity further. These components jointly define a prior GMM and regularize the posterior estimation of the GMM on the target data. We demonstrated the superior performance of Praxis-BGM over competing deterministic classification-based methods in both simulation studies and real-world applications, as well as the advantage of incorporating prior knowledge over purely unsupervised clustering. In the context of breast cancer, Praxis-BGM successfully transferred subtype-specific component structures from METABRIC to TCGA-BRCA, yielding significant improvements in biological relevance and clinical utility while also enabling the discovery of cluster-associated genes. We further showed that Praxis-BGM can flexibly integrate multi-omic layers for in-serial analysis by using cluster-specific priors derived from a preceding omic layer to inform the analysis of the next layer. We also demonstrated that the application of Praxis-BGM extends naturally to cell-type label transfer in single-cell transcriptomics to effectively leverage annotated reference datasets to simultaneously cluster and annotate query cells.

When multiple omics layers are available, the choice of which layer to prioritize for transfer learning, and whether a downstream in-serial update with additional omics layers is beneficial, depends mainly on two considerations: (i) biological relevance to the scientific question and study objective, and (ii) data availability and completeness. For example, in the breast cancer bulk transcriptomics application, our primary objective and main validation metric were to recover PAM50 labels in the target data. Accordingly, the transcriptomic layer is naturally the most informative layer for this objective, whereas the miRNA layer may offer complementary refinement for explorative analysis. By contrast, other omics layers, such as copy number variation (CNV), may not be prioritized for this specific goal. In practice, it is also often reasonable to begin with the layer measured in the largest and most complete set of subjects. More broadly, Praxis-BGM is a highly flexible approach, as priors may be derived from an external source dataset in the same feature space, from another omics layer measured in the same cohort, or from a sequential combination of both, as illustrated in the breast cancer bulk transcriptomics application. The most appropriate choice of modeling strategy depends on the research objective, the underlying biology, and the structure and availability of the source and target data. Moreover, when the source dataset contains high-quality integrative cluster labels estimated from multi-omics data, Praxis-BGM can still leverage that information to perform transfer learning using a single omics layer shared between the source and target datasets; the target dataset does not need to contain all omics layers available in the source data. An important direction for future work is the development of a parallel multi-omics transfer-learning framework that jointly integrates multiple omics layers while allowing layer-specific priors to be derived from potentially distinct source datasets.

One caveat about statistical transfer learning is the risk of negative transfer, which could occur when the knowledge transferred from the source data reduces the performance of modeling and inference in the target data (Pan and Yang 2010). Inappropriate priors from unsuitable source data for Praxis-BGM can also potentially lead to negatively biased posteriors. Our framework seeks to mitigate this potential negative impact by reconciling discrepancies between priors and data through variational inference. Simulation studies demonstrate that Praxis-BGM maintains strong performance even under severely mis-specified priors. Future work could focus on enhancing robustness by learning the reliability of priors derived from multiple sources and selecting the most informative source datasets or samples through data-driven methods. For supervised statistical transfer learning methods, well-established strategies exist to select the set of informative source samples (Li *et al.* 2022). Wycoff *et al.* (2025) introduced a prior-misalignment-risk minimizer conditional on source parameters that estimates the inclusion probability of each source dataset through a full Bayesian framework. A similar mechanism could be incorporated into the Praxis-BGM framework to reduce the risk of negative transfer.

## Supplementary Material

btag395_Supplementary_Data

## Data Availability

The METABRIC breast cancer dataset (Curtis *et al.* 2012) was accessed through cBioPortal (Cerami *et al.* 2012, Gao *et al.* 2013, de Bruijn *et al.* 2023), and the TCGA-BRCA dataset (Network 2012) was downloaded from the R package curatedTCGAData (Ramos *et al.* 2020). The pancreas scRNA-seq benchmark data followed the study of Luecken *et al.* (2022). The benchmark datasets are publicly available at https://doi.org/10.6084/m9.figshare.12420968.v8, where the authors assembled datasets from the GEO and ArrayExpress repositories listed in the article’s Supplementary Materials.
